# Automated versus manual urine output monitoring in the intensive care unit

**DOI:** 10.1038/s41598-021-97026-8

**Published:** 2021-08-31

**Authors:** Joni Minor, Ali Smith, Frederic Deutsch, John A. Kellum

**Affiliations:** 1 University of Pittsburgh Medical Center (UPMC), Pittsburgh, PA USA; 2 Department of Critical Care Medicine, Center for Critical Care Nephrology and CRISMA Center, University of Pittsburgh, 3347 Forbes Avenue, Suite 220, Pittsburgh, PA 15213 USA; 3 Biostatistics Statistical Consulting Ltd, Mercaz Renanim Maccabim, Israel

**Keywords:** Nephrology, Outcomes research

## Abstract

Acute kidney injury (AKI) is defined by changes in serum creatinine and urine output (UO). Significant limitations exist regarding accurate ascertainment of urine output even within the intensive care unit. We sought to evaluate an automated urine output collections system and compare it to nursing measurements. We prospectively collected urine output using an electronic urine monitoring system and compared it to charted hourly UO in 44 patients after cardiac surgery at a single university hospital ICU. We calculated UO and oliguria rates and compared them to data from the sensor and from nursing charting. A total of 187 hourly UO measurements were obtained and on average, UO was reported 47 min late, with a median of 18 min, and a maximum of almost 6 h. Patients had a mean hourly UO of 76.3 ml over the observation period. Compared to manual measurements by study personnel, nurses significantly overestimated hourly UO by 19.9 ml (95% CI: 10.3; 29.5; *p* =  < 0.001). By contrast, the mean difference between the UO measured with the sensor and by study personnel was 2.29 ml (95% CI: − 6.7; 11.3), *p* = 0.61. Electronic UO monitoring is significantly more accurate than nurse-performed manual measurements in actual intensive care patients. Furthermore, timely ascertainment of UO is difficult to achieve with manual technique, resulting in important delays in detecting oliguria perhaps leading to missed cases of AKI.

Acute kidney injury (AKI) is defined by an abrupt decrease in renal function and currently is assessed by changes in serum creatinine and urine output (UO). While both markers have serious limitations^1^, their combination provides more information than either alone^2^. In critically ill children, assessment of AKI according to the serum creatinine alone failed to identify AKI in 67.2% of those patients with low UO^3^. Similarly, in adults, serum creatinine misses more than a third of cases of AKI in the ICU that can be detected using oliguria^2^. Interestingly, stage 3 AKI by UO alone is associated with a higher hospital mortality than stage 3 by serum creatinine alone (17.7% vs. 11.6%)^2^. In a recent study including 6637 patients undergoing cardiac surgery, more than 40% developed isolated oliguria (no AKI based on serum creatinine criteria)^4^. Major adverse kidney events (MAKE) at 6 months increased in these patients from 4.5 to 7.6% (*p* =  < 0.01). Even stage 1 AKI by oliguria alone (i.e. < 12 h of oliguria and no AKI by creatinine criteria) was associated with an increased risk of MAKE at 6 months (odds ratio, 1.76; 1.20–2.57; *p* = 0.004), mainly driven by persistent renal dysfunction (odds ratio, 2.01; 1.26–3.18; *p* = 0.003)^4^. Moreover, the highest mortality is seen in patients with both azotemia and oliguria^2,4^.

In recent years, inter-assay variation for serum creatinine has been markedly reduced using isotope dilution mass spectrometry-traceable standards. Similarly, precision for measurement of various vital signs (e.g. heart rate, blood pressure, oxygen saturation) has been improved with automated systems that are not susceptible to interobserver variation. These measurements are also transferred electronically to electronic medical record (EMR) systems ensuring data capture. Conversely, UO measurement remains archaic and inaccurate and data capture within the EMR is highly variable. Jin and coworkers compared intensive UO monitoring, defined as hourly recordings and no gaps > 3 h to non-intensive monitoring in more than 15,000 critically ill patients^5^. They found that intensive monitoring was associated with improved survival among patients experiencing AKI. With or without AKI, patients with intensive monitoring also had less fluid volume. Oliguria for three or more days is an independent predictor for the development of complications including sepsis following AKI^6^.

Electronic UO monitoring has already been shown to be accurate in a precisely controlled in-vivo setting^7^. Our goal with this prospective observational study was to compare an electronic real-time UO monitoring device with standard manual technique in terms of accuracy and efficiency in a real-life clinical setting where bedside clinicians were blinded to the data from the device.

## Methods

We conducted a prospective observational study comparing the use of the Clarity RMS (RenalSense, Jerusalem, Israel) electronic urine monitoring system to manual recording of a hospitalized patient’s hourly UO. This study was approved by the University of Pittsburgh’s Institutional Review Board (IRB#: PRO1703045), and all methods were performed in accordance with the University’s guidelines and regulations. The device was used on patients with Foley catheters in place cared for in a single university hospital ICU. We selected the cardiothoracic surgery ICU because hourly UO measurement is the standard of care in this unit. During the study, the Clarity RMS console was blinded to the clinicians. Study coordinators monitored UO by weighing the urine collection bag via a digital scale and this was used the as gold standard for comparisons. Coordinators also abstracted hourly UO recordings obtained by the bedside nurses during a 4–6-h period and finally, they downloaded electronic UO data from the Clarity RMS system over the same period of time. In order to prevent interference with the nursing measurement of UO, the bag was placed inside of a small container and placed on a digital scale. All scales were zeroed before the first measurement was recorded. Placement of the container and support of tubing and a uniform style of measuring between the nurses was maintained as much as possible. Each hourly measurement was recorded as a delta from the measurement in the previous hour. Should the bag need emptying from the drainage tap, the “first hour” was recorded again after emptying. To ensure the bedside nurse remained blinded to the actual purpose of the study, the study coordinators recorded all nursing care related to the Foley and various other nursing care tasks. In addition, the sensor kits were connected to the console and the screen was covered to ensure no data from the device was available for viewing by the nursing staff. At the end of the shift, an investigator abstracted the nursing documentation regarding hourly urine output from the EMR while the study coordinator downloaded the device data onto a study USB drive. Measurements of patient UO inserted in their respective time slots in the EMR were compared to their time stamp record indicating when the UO was actually recorded into the EMR system. “Late” measurements were defined according to the time difference between the UO time slot and the “real time” indicated by the time stamp. Data were entered into a secure database maintained by the study project manager.

### Data management

We removed any UO recordings that were based on less than 15 min of data or any hours that could not be calculated due to missing information from the scale (the gold standard). For incomplete collections, we extrapolated UO recorded for the full hour assuming linearity. For example, if 50 ml were recorded during only 40 min, the UO analyzed was 50/40*60 = 75 ml. Analysis for the sensor validation and comparison to the scale data and nursing measurements consisted of 187 hourly UO measurements from 44 patients. Nursing actions were observed during day shifts. All patient data were anonymized prior to analysis.

### Statistical analysis

**Figure 1:**
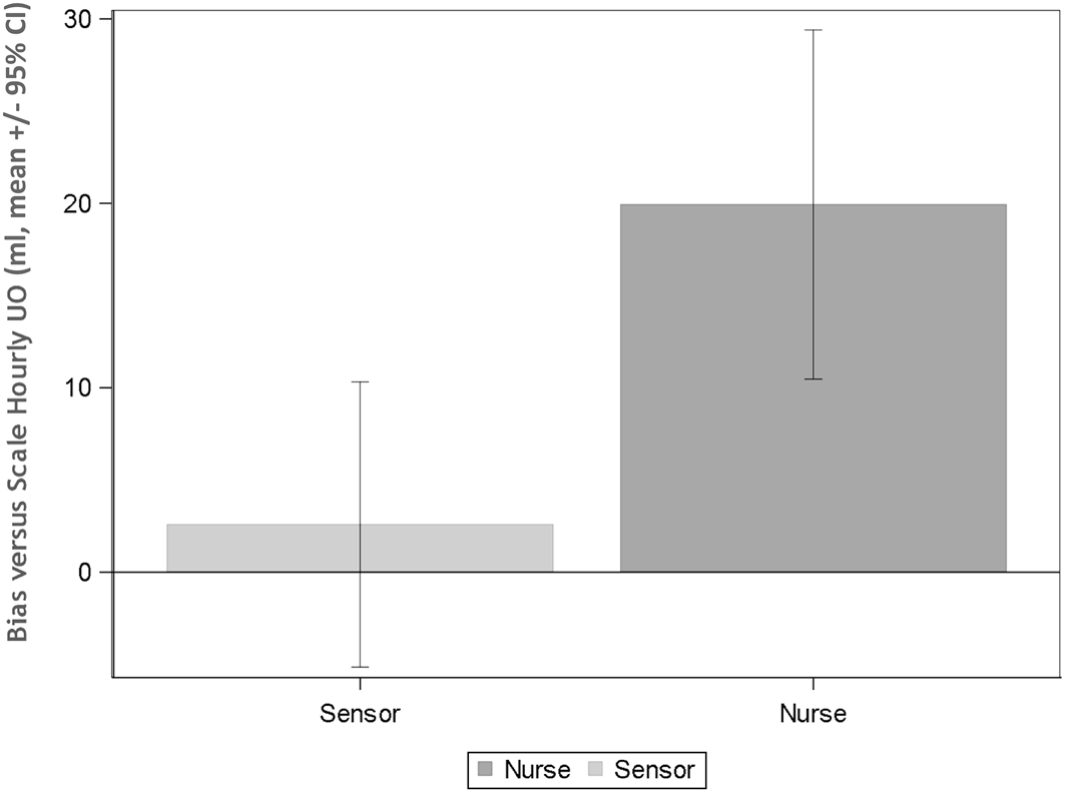
Comparison between manual and automated. Comparison of Urine output documented in the electronic medical record by the bedside nurse and sensor measurements to the hourly scale measurements (gold standard) by study personnel.

The percent of missing measurements of UO recorded by the two modalities (nurses reading and sensor) is presented with the 95% binomial Confidence Interval (CI). Descriptive statistics of the difference (bias) in hourly UO measured by the modalities and with the scale are presented (Fig. 1). Repeated measures ANOVA model were used to estimate the mean bias, its standard deviation and 95% CI, in order to take into consideration within patient correlation. Model estimated means (LSmeans) with 95% CI’s and level of significance are presented for the nurses and the sensor, as well as the difference between them. Means and standard deviation (SD) are also provided. Statistical analyses were performed in SAS v9.4 (SAS institute, Cary, NC, USA), nominal p-values and non-adjusted CI are presented.

### Ethics approval and consent to participate

This study was approved by the University of Pittsburgh’s Institutional Review Board (IRB#: PRO1703045).

### Consent for publication

Participating individuals signed an Informed Consent Form approved by the University of Pittsburgh’s Institutional Review Board (IRB#: PRO1703045).

## Results

### Patients

In total, 187 h were observed from 44 patients. All patients underwent cardiac surgery and were observed in the first 24 h following the procedure. Because the focus of the study was on urine output measurement and nursing care, patient information was not collected. Hourly UO measurements using the three methods are shown in Table 1.

**Table 1:** Hourly urine output assessed by three methods.

	N	Mean	SD	Min	Median	Max	Missed (%)*
Scale	187	76.34	72.71	0.00	53.20	496.0	NA
Nurse	114	139.3	111.2	5.00	120.0	750.0	39%
Sensor UO-normalized	171	83.52	65.78	4.00	66.00	356.0	8.6%

N indicates the number of patient-hours included. SD, standard deviation. NA is not applicable. *Percent of hours where no value was recorded.

### Manual measurements

Out of the 187 hourly UO measurements available, 73, 39.0% (95% CI: 32.0%; 46.4%) were missing a bedside nurse recording. On average, the UO was reported 47 min late, with a median of 18 min, and a maximum of almost 6 h. Over the observation period, the mean hourly UO for all patients together was 76.3 ml ± 72.71 SD. On average the nurses significantly overestimated the hourly UO by 19.9 ml (SD: 44.0 ml, 95% CI: 10.3; 29.5; *p*-value < 0.001).

### Automated measurement

Out of the 187 hourly UO measurement available, 16, 8.6% (95% CI: 5.0%; 13.5%) were missing. Out of the 187 hourly urine outputs measured with the sensor, 6 were partial (the sensor didn’t measure the UO for the whole hour). The mean difference between the UO measured with the sensor and with the scale was 2.29 ml (SD: 34.0 ml, 95% CI: − 6.7; 11.3), a difference that was not statistically significant, *p*-value = 0.61.

### Comparison between manual and automated

The mean measurement bias between the sensor and the scale was significantly lower than the mean bias between the nurse and the scale by 17.3 ml (95% CI: 7.0; 27.7; *p*-value: < 0.01) on average (model estimated mean difference, Fig. 1). Furthermore, there were 6 patients who had oliguria (< 0.5 ml/kg/hr) for at least one hour that were missed by the bedside nurse but detected by the sensor including one patient who met UO criteria for AKI. In 4 additional patients, the oliguric episode was detected sooner by the sensor (1 h in 2 patients, 2 h and 5 h in others).

### Observed nursing actions

Nursing actions were observed for 44 patients yielding a total of 236 observed hours. The observed hours per case ranged from 1 to 7 h (median 6 h). Out of 236 h, the nurses practiced standard precautions of hand hygiene and/or donning gowns ranged between 0.5 to 7.0 times per hour observed (median 2.0). Out of the 44 cases studied, hourly UO was recorded in real time, in the EMR, a median of 50% of the observed hours per case; the rest of the hourly urine output measurements in the patient’s EMR were filled retroactively by the nurse. The urinometer was touched and emptied each time the urine output measurement was recorded; i.e., nursing contact with the urine bag occurred once every two hours.

## Discussion

The majority of physiological parameters measured in patients in a critical care setting are electronically monitored. Automation not only reduces workload and human error, but also provides alarms and warnings when these parameters fall outside a pre-set range. UO may be the most vital physiological parameter that still involves manual recordings in the critical care setting^8^. Our results indicate that an automated system performs considerably better than manual methods to monitor UO. Manual recordings were frequently (39%) missed, often late, and were off by more than 17 ml/hr. For a 70 kg patient, the criteria for stage 1 or stage 2 AKI by UO is 35 ml/hr (for 6 or 12 h respectively). A bias of 17 ml approaches 50% of the threshold value. The patients in this study made 76.3 ml/hr of UO on average. A bias of 17 ml is 22.3% of the total UO. Thus, the accuracy provided by manual monitoring of UO may be insufficient to detect AKI in some patients. Indeed, oliguria detected by the sensor was missed in 6 patients including 1 who met AKI criteria and detected late in 4 others by the bedside nurses. Even in the absence of AKI, UO monitoring is essential for critically ill patients as oliguria is an early predictor of mortality in these patients^9^.

Even if the accuracy of manual UO monitoring could be improved, there is still the problem of workflow. For many patients, care needs make it simply impossible to provide on-time UO measurements each hour. As such manual UO monitoring often includes values recorded as an average over time and not consecutive measurements^10^. In our study, 73/187 (39%) hourly measures were missed by the bedside nurses compared to only 16/187 (8.6%) by the device. Another study compared intensive UO monitoring (hourly measurements with no gaps > 3 h) with less intensive monitoring for the first 48 h after ICU admission in 15,724 adults^5^. Intensive monitoring of UO was only achieved in 4,049 patients (26%) and significantly more AKI was detected in these patients (OR, 1.22; *P* < 0.001). After adjustment for age and severity of illness, intensive UO monitoring was associated with improved survival but only among patients experiencing AKI. With or without AKI, patients with intensive monitoring also had less cumulative fluid volume (2.98 L vs 3.78 L; *p* =  < 0.001) and less fluid overload (2.49% vs 5.68%; *p* =  < 0.001) over the first 72 h of ICU stay^5^.

A second issue is the manual UO measurement procedure itself. One of the most important changes in patient care made over the last 25 years relates to changes in hygiene protocols for many intensive care patients^11^. Especially with diseases like COVID-19, donning isolation gowns and masks and intensive hand hygiene are part of standard precautions for extensive effort to reduce the spread of infection. Real-time contact-less patient monitoring of vital signs provides continuous monitoring of patient status as well as an additional resource for reducing added exposure to patient bodily fluids, excretions and secretions^12,13^. Electronic real-time monitoring is not only essential for vital sign monitoring but also would provide an added reduction of patient contact and urine bag manipulation required of manual measurements.

Importantly, our study involved only 44 patients with a mean observation period of 4.25 h per patient. However, given very large differences seen between standard of care and the sensor compared to the gold standard, it is unlikely that a sample would have changed our results. Furthermore, we only made observations during daylight hours. Given reduced staffing at night, the standard of care may well have even been less acurate. Another limitation to our study was that we did not collect detailed patient-level data appart from UO. As such we cannot determine the reasons for inaccuracy with manual collection or if the sensor improved AKI detection since some patients may have met serum creatinine criteria. Finally, our gold standard using a scale would not be as accurate in patients with very concentrated urines. This is not the case in post-operative heart surgery patients but results with this method could vary in other circumstances.

## Conclusion

In conclusion, electronic UO monitoring is significantly more accurate than measurements taken by the bedside nurses in actual intensive care patients. Furthermore, timely ascertainment of UO is difficult to achieve with manual technique resulting in important delays in detecting oliguria perhaps resulting in missed cases of AKI.

## Data Availability

The datasets used and analyzed during the current study are available from the corresponding author on reasonable request.

## Abbreviations

AKI: Acute kidney injury
UO: Urine output
ICU: Intensive care unit
MAKE: Major adverse kidney event
EMR: Electronic medical record
CI: Confidence interval
